# Supplementary material for: The Effectiveness of eHealth Interventions in Promoting the Health Literacy of Informal Caregivers: A Systematic Literature Review Protocol
Source: Healthcare (Basel). 2024 Nov 25;12(23):2354. doi: 10.3390/healthcare12232354 (PMC11641513; doi:10.3390/healthcare12232354)
Supplement: Supplementary file 1 [file healthcare-12-02354-s001.zip › File S2.Search Strategy in the Scopus Database.pdf]

Search Strategy in the Scopus Database

Database	Search strategy/query	Number of records	Date of search
Scopus	(Informal Caregiver*) AND TITLE-ABS-KEY(digital* OR (digital health) OR e-health OR ehealth OR technology* OR chatbot OR (assistent virtual) OR (artificial intelligence) OR (virtual agent) OR (virtual assistent) OR (conversational agent) OR (Biomedical Technology) OR (Internet Utilization Online) OR web OR mHealth OR eHealth OR mobile OR application OR computer OR smartphone OR (access to information) OR (health communication) OR digital OR ehealth OR electronic OR mhealth OR mobile OR technolog* OR computer OR internet OR tele* OR online) AND TITLE-ABS-KEY((health-education) OR (health-information) OR (health literacy) OR (health promotion) OR (patient education) OR (health knowledge) OR (health behavior) OR (Computer Literacy) OR (Computer Literacies) OR (ehealth literacy) OR (e-health literacy) OR (digital literacy) OR (digital health literacy) OR (mhealth literacy) OR (m-health literacy) OR (telehealth literacy) OR (tele-health literacy) OR (information literacy)OR eHEALS OR (eHealth literacy scale) OR (e-HLS) OR (electronic health literacy scale) OR (digital Health Literacy Instrument) OR (eHealth readiness scale))	605	23/09
